# Antioxidant Systems are Regulated by Nitric Oxide-Mediated Post-translational Modifications (NO-PTMs)

**DOI:** 10.3389/fpls.2016.00152

**Published:** 2016-02-16

**Authors:** Juan C. Begara-Morales, Beatriz Sánchez-Calvo, Mounira Chaki, Raquel Valderrama, Capilla Mata-Pérez, María N. Padilla, Francisco J. Corpas, Juan B. Barroso

**Affiliations:** ^1^Group of Biochemistry and Cell Signaling in Nitric Oxide, Department of Experimental Biology, Center for Advanced Studies in Olive Grove and Olive Oils, Faculty of Experimental Sciences, University of JaénJaén, Spain; ^2^Group of Antioxidants, Free Radicals and Nitric Oxide in Biotechnology, Food and Agriculture, Department of Biochemistry, Cellular and Molecular Biology of Plants, Estación Experimental del Zaidín, Consejo Superior de Investigaciones CientíficasGranada, Spain

**Keywords:** ascorbate-glutathione cycle, catalase, superoxide dismutase, peroxiredoxin, nitric oxide, *S*-nitrosylation, tyrosine nitration

## Abstract

Nitric oxide (NO) is a biological messenger that orchestrates a plethora of plant functions, mainly through post-translational modifications (PTMs) such as *S*-nitrosylation or tyrosine nitration. In plants, hundreds of proteins have been identified as potential targets of these NO-PTMs under physiological and stress conditions indicating the relevance of NO in plant-signaling mechanisms. Among these NO protein targets, there are different antioxidant enzymes involved in the control of reactive oxygen species (ROS), such as H_2_O_2_, which is also a signal molecule. This highlights the close relationship between ROS/NO signaling pathways. The major plant antioxidant enzymes, including catalase, superoxide dismutases (SODs) peroxiredoxins (Prx) and all the enzymatic components of the ascorbate-glutathione (Asa-GSH) cycle, have been shown to be modulated to different degrees by NO-PTMs. This mini-review will update the recent knowledge concerning the interaction of NO with these antioxidant enzymes, with a special focus on the components of the Asa-GSH cycle and their physiological relevance.

## Introduction

Nitric oxide is a gaseous molecule and a pivotal biological messenger. NO is involved in signaling pathways that are related to fundamental processes in plant biology such as growth and development (Beligni and Lamattina, 2000; Pagnussat et al., 2002), senescence (Begara-Morales et al., 2013) and response to abiotic (Corpas et al., 2011; Siddiqui et al., 2011) or biotic stress (Delledonne et al., 1998; Durner et al., 1998; Feechan et al., 2005). Generally, the rise in NO levels in response to stress conditions is accompanied by another group of molecules called reactive oxygen species (ROS), some of which, particularly H_2_O_2_, are also involved in multiple signaling pathways (Neill et al., 2002). This mini-review will explore recent findings concerning the modulation of the main antioxidant enzymes by NO, especially the enzymatic components of Asa-GSH cycle, with particular attention to the molecular mechanism underpinning this key regulatory pathway in response to stress situations.

## Nitric Oxide-Mediated Post-Translational Modifications: Nitration and *S*-Nitrosylation

Nitric oxide mainly transmits its action via post-translational modifications, such as *S*-nitrosylation and tyrosine nitration, which can regulate the function of the target proteins (Astier and Lindermayr, 2012). These NO-PTMs may be involved in cell signaling under physiological and stress conditions (Corpas et al., 2015).

Tyrosine nitration, which is mediated mainly by peroxynitrite (ONOO^-^), consists of the addition of NO_2_ radicals to one of the two equivalent ortho-carbons of the aromatic ring of tyrosine residues leading to 3-nitrotyrosine (Gow et al., 2004; Radi, 2004). This modification converts the tyrosine into a negatively charged residue and causes a marked shift in the hydroxyl group’s pKa (Turko and Murad, 2002; Abello et al., 2009) which can affect the target proteins resulting in a gain, loss or no change in the protein’s function (Souza et al., 2008; Radi, 2013). Although tyrosine nitration has been traditionally considered as an irreversible mechanism and a nitrosative stress marker, the existence of tyrosine denitrase activity that reduces 3-nitrotyrosine in mammalian cells (Görg et al., 2007; Deeb et al., 2013) pointing toward a role of tyrosine nitration in NO-mediated signaling processes in these cells.

*S*-nitrosylation consists of the addition of a NO group to a cysteine thiol leading to *S*-nitrosothiols (SNOs) and consequently can also alter the function of a broad variety of proteins (Hess et al., 2005; Astier et al., 2011). *S-*nitrosoglutathione (GSNO), formed by *S*-nitrosylation of the antioxidant GSH, is the major low-molecular-weight *S-*nitrosothiol. It is considered to be a NO reservoir in cells (Gaston et al., 1993; Durner et al., 1999; Leitner et al., 2009) that due to its phloem mobility is involved in signaling mechanisms. Furthermore, GSNO can mediate transnitrosylation reactions in which a new *S*-nitrosothiol is generated by transferring its NO group to a new cysteine thiol group (Hess et al., 2005).

*S-*nitrosylation is a reversible mechanism since SNO can be specifically and enzymatically broken down by thioredoxins (Benhar et al., 2008; Kneeshaw et al., 2014), in addition to the non-enzymatic decomposition by antioxidants such as ascorbate or glutathione. Furthermore, *S*-nitrosoglutathione reductase (GSNOR) decomposes GSNO and indirectly controls SNO levels (Liu et al., 2001; Feechan et al., 2005).

In recent years, mounting evidence has shown that SNOs are fundamental players in NO-signaling pathways in plant biology (Belenghi et al., 2007; Romero-Puertas et al., 2007, 2008; Lindermayr and Durner, 2009; Astier et al., 2011; Hu et al., 2015), with an important role in plant immunity and plant response to abiotic stresses (Feechan et al., 2005; Rusterucci et al., 2007; Valderrama et al., 2007; Corpas et al., 2008; Chaki et al., 2009a, 2011a,b). Due to its importance, increased efforts have been made to identify the processes that could be regulated by SNOs and subsequently hundreds of proteins that undergo *S-*nitrosylation under physiological or adverse conditions have been identified over the past decade.

## *S*-Nitrosylation Controls ONOO^-^ Levels via Regulation of PrxII E

Peroxiredoxins (Prx) are thiol based peroxidases that can be involved in multiple functions in addition to its role in detoxifying H_2_O_2_ (for review see Bhatt and Tripathi, 2011). Some Prxs have been identified to be regulated by NO-PTMs in animals and plants. In mammals, *S-*nitrosylation inhibits the enzymatic activity of neuronal Prx2 (Fang et al., 2007) and Prx1 (Engelman et al., 2013) whereas the peroxidase activity of Prx2 from mammalian erythrocytes was induced after tyrosine nitration (Randall et al., 2014). In plants, *S-*nitrosylation inhibits the peroxidase activity of PrxII E (Romero-Puertas et al., 2007) and PrxII F (Camejo et al., 2015). Interestingly, some members of Prx family posses ONOO^-^ reductase activity (Bryk et al., 2000; Romero-Puertas et al., 2007; Pedrajas et al., 2010) and therefore could protect against ONOO^-^-mediated oxidative and nitrosative stresses. In plants, PrxII E is *S-*nitrosylated during hypersensitive response (Romero-Puertas et al., 2008) and this modification inhibits its peroxynitrite reductase activity promoting tyrosine nitration (Romero-Puertas et al., 2007). Therefore, *S-*nitrosylation emerges as a key mechanism in ONOO^-^ homeostasis, regulating endogenous level of ONOO^-^ and tyrosine nitration via control of PrxII E (Romero-Puertas et al., 2007). Changes in ONOO^-^ levels and/or tyrosine nitration have been related to several abiotic/biotic stresses (Valderrama et al., 2007; Corpas et al., 2008; Chaki et al., 2009a, 2011a,b). Consequently, understanding if *S-*nitrosylation of PrxII E could be involved in plant response to these stress conditions is a good issue to be addressed in the future.

## Nitric Oxide Interactions with Catalase and Superoxide Dismutases

Superoxide dismutase (SOD) is a group of metalloenzymes that catalyze the disproportionation of superoxide radicals into H_2_O_2_ (Fridovich, 1986; Halliwell and Gutteridge, 2000). SODs are classified into three main types containing Mn, Fe, or Cu plus Zn as prosthetic metals and they are present in all cell compartments (Parker et al., 1984; Zelko et al., 2002). In eukaryotic cells from different organisms, it has been demonstrated that Mn-, Fe-, and CuZn-SODs undergo inactivation by peroxynitrite-mediated nitration (Demicheli et al., 2007; Martinez et al., 2014) and SOD activity is increased after GSNO treatment (Sehrawat et al., 2013). Recently, *in vitro* approaches have been used to analyze the effect of NO-mediated PTMs on the different SOD isozymes in *Arabidopsis thaliana*. Thus, whereas *S-*nitrosylation did not affect SOD activities, nitration inhibited Mn-SOD1, Fe-SOD3, and CuZn-SOD3 activity to different degrees but affected no other SOD isozymes (Holzmeister et al., 2015).

On the other hand, catalase, which is a peroxisomal key enzyme that regulates H_2_O_2_ levels (Chance et al., 1979; Kirkman and Gaetani, 1984), was one of the first antioxidant enzymes to be analyzed *in vitro* to check how its activity can be modulated by NO donors (Clark et al., 2000). At present, it is known that plant catalase can be nitrated and *S*-nitrosylated *in vitro*, both of which inhibit its activity (Clark et al., 2000; Ortega-Galisteo et al., 2012), although, according to the literature available, the specific target residues have not yet been identified. Very recently, it has been determined by proteomic approaches that catalase undergoes increasing nitration during pepper fruit maturation, decreasing its activity as consequence of potential tyrosine nitration as corroborated after treatment with SIN-1 (a peroxynitrite donor; Chaki et al., 2015). This inhibition could imply a lower capacity for removing H_2_O_2_ and therefore is well correlated with the increase of the oxidative metabolism observed during this physiological process (Martí et al., 2011; Chaki et al., 2015).

## Ascorbate-Glutathione Cycle and Nitric Oxide-PTMs

Ascorbate-glutathione cycle is a pivotal antioxidant system involved in the regulation of H_2_O_2_ levels (Asada, 1992; Noctor and Foyer, 1998; Shigeoka et al., 2002) under development and unfavorable conditions in plant cells. The cycle is composed of the enzymes APX, MDAR, DHAR, and GR plus the non-enzymatic antioxidants ascorbate and glutathione (GSH). Concomitant to H_2_O_2_ reduction to water, APX catalyzes the oxidation of ascorbate to monodehydroascorbate (MDA) which can spontaneously generate dehydroascorbate (DHA). Ascorbate is regenerated by MDAR and DHAR using NADPH and GSH as electron donors, respectively. Finally, GR is involved in regenerating GSH levels.

**FIGURE 1:**
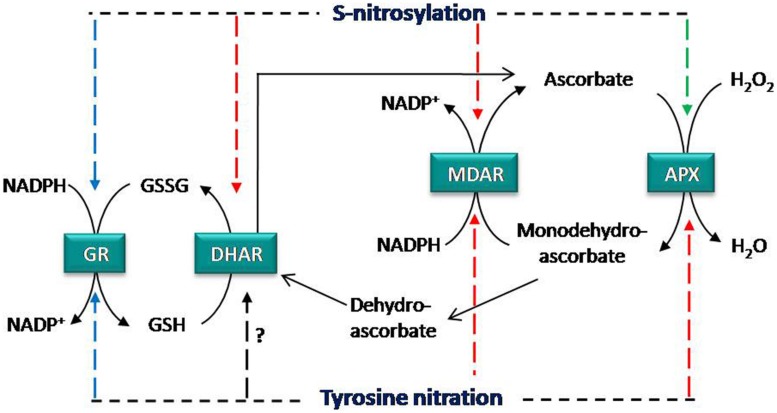
**Regulation of ascorbate glutathione cycle by NO-PTMs.** APX activity is inhibited by tyrosine nitration and enhanced by *S-*nitrosylation, whereas MDAR is inhibited by both NO-PTMs. DHAR is inhibited by *S-*nitrosylation and GR is not affected by these PTMs. Color of arrows shows the effect of tyrosine nitration and *S-*nitrosylation on enzymatic activities; red: inhibition, green: enhancement, and blue: no effect. APX, ascorbate peroxidase; MDAR, monodehydroascrobate reductase; DHAR, dehydroascorbate reductase; GR, glutathione reductase.

Analyzing how NO regulates Asa-GSH cycle is a key issue to understand the interplay between NO and antioxidant systems (**Figure 1**). In this sense, enzymatic activity of the components of Asa-GSH cycle can be modulated by NO and under different stress situations (Groβ et al., 2013). Additionally, these enzymes have been identified as targets of NO-PTMs, identifying in some cases the molecular mechanism involved in these modifications (**Table 1**).

**Table 1:** Effect of NO-PTMs on components of Asa-GSH cycle.

Protein	NO-PTM	Effects	Target	Plant species	Reference
Ascorbate peroxidase (APX)	Tyrosine nitration	Decreased activity	Tyr235^(1)^	*Pisum sativum*	Begara-Morales et al., 2014
	*S-*nitrosylation	Increased activity	Cys32^(1)(2)(3)^	*Arabidopsis thaliana,*	Begara-Morales et al., 2014;
				*Pisum sativum*	Yang et al., 2015
Monodehydro-ascorbate reductase (MDAR)	Tyrosine nitration	Decreased activity	Tyr345^(1)^	*Pisum sativum*	Begara-Morales et al., 2015
	*S-*nitrosylation	Decreased activity	Cys68^(3)^	*Pisum sativum*	Begara-Morales et al., 2015
Dehydro-ascorbate reductase (DHAR)	Tyrosine nitration	N.D.	N.D.	N.D.	Fares et al., 2011; Kato et al., 2013; Puyaubert et al., 2014
	*S-*nitrosylation	Decreased activity	Cys20^(1)(2)^,	*Arabidopsis thaliana;*	
			Cys147^(1)(2)^	*Solanum tuberosum*	
Glutathione reductase (GR)	Tyrosine nitration	No effect	N.D.	*Pisum sativum*	Begara-Morales et al., 2015
	*S-*nitrosylation				


*Nitration and *S-*nitrosylation targets have been identified by different technological approaches: (1) Mass spectrometry, (2) site-directed mutagenesis, and (3) *in silico* identification. ND: Not determined.*

### Regulation of Asa-GSH Cycle by Tyrosine Nitration

Proteomic approaches have identified all enzymes of the Asa-GSH cycle as potential nitrated proteins (Chaki et al., 2009b; Lin et al., 2012; Tanou et al., 2012). However, information related to the specific impact of this modification on the structure of these target proteins and the role of the tyrosine target of nitration is necessary in order to understand the cross-talk between NO and ROS in the antioxidant defense against nitrosative stress. In this respect, two recent studies have identified the tyrosine target(s) of nitration and its (their) potential role within the mechanistic activity of the Asa-GSH cycle enzymes, showing that this NO-PTM could compromise the Asa-GSH cycle functioning (Begara-Morales et al., 2014, 2015). Pea cytosolic APX is inactivated by ONOO^-^ as consequence of tyrosine nitration (Begara-Morales et al., 2014) and as result the detoxification of H_2_O_2_ by Asa-GSH cycle could be compromised (**Figure 1**). Proteomics and *in silico* approaches identified the Tyr235 as the most reliable target responsible for APX inactivation, since this residue is located just at 3.6 Å from the heme group at the bottom of the catalytic pocket (Patterson and Poulos, 1995; Jespersen et al., 1997; Mandelman et al., 1998; Begara-Morales et al., 2014). Consequently, Tyr235 nitration may disrupt heme-group properties and result in a loss of activity (Begara-Morales et al., 2014).

Monodehydroascorbate reductase, which is involved in the regeneration of ascorbate (**Figure 1**), is another significant modulation point of the Asa-GSH cycle. In this case, ONOO^-^ mediates nitration of recombinant pea MDAR at Tyr213, Tyr292, and Tyr345, causing an inhibition of the enzymatic activity (Begara-Morales et al., 2015), and therefore may disrupt the regeneration of ascorbate and compromise the functioning of the Asa-GSH cycle. Site-directed mutagenesis demonstrates that Tyr345 is the main residue responsible for the loss of activity after nitration, since this tyrosine is located just at 3.3 Å from His313, which is involved in NADP binding, suggesting that the nitration of this tyrosine could alter the positioning of the cofactor, thereby decreasing protein activity (Begara-Morales et al., 2015). DHAR is the other enzyme involved in the regeneration of ascorbate, but, although DHAR has been reported to be nitrated (Tanou et al., 2012) and its activity modulated by NO (see Groβ et al., 2013), no information is available on the tyrosine(s) involved in this modification and the impact on the protein structure.

Glutathione reductase has also been identified as tyrosine nitration target (Chaki et al., 2009b). In animals, peroxynitrite inhibits human and bovine GR activity by nitration of Tyr106 and Tyr114 which are located close to the GSSG binding zone (Francescutti et al., 1996; Savvides et al., 2002). However, very recently and in contrast to animals, it has been strikingly shown that chloroplastic and cytosolic pea GR activities are not affected by peroxynitrite-mediated tyrosine nitration (Begara-Morales et al., 2015). This behavior is unusual in higher plants, where the main effect of tyrosine nitration on target proteins is usually a loss of function (Astier and Lindermayr, 2012; Begara-Morales et al., 2013; Chaki et al., 2013; Corpas et al., 2013).

### *S*-Nitrosylation on Asa-GSH Cycle

*S-*nitrosylation has emerged as a key PTM that regulates fundamental processes in plant biology such as plant immunity or plant response to (a)biotic stress. Consequently, this PTM has become the main pathway by which NO acts as a signaling molecule. Hundreds of proteins related to a wide range of metabolic pathways have been reported to be *S*-nitrosylated in plants. For instance, NO regulates many enzymes involved in ROS/RNS generation/scavenging such as GSNOR (Frungillo et al., 2014), NADPH oxidase (Yun et al., 2011), catalase (Ortega-Galisteo et al., 2012), and peroxiredoxinII E (Romero-Puertas et al., 2007) and II F (Camejo et al., 2015). *S-*nitrosylation appears to be critical to GSNO and ONOO^-^ homeostasis as this NO-PTM inhibits GSNOR and PrxII E activities (Romero-Puertas et al., 2007; Frungillo et al., 2014) that decompose GSNO and ONOO^-^, respectively. Consequently, during a nitro-oxidative stress the inactivation of these enzymes by *S-*nitrosylation could favor the accumulation of these NO-derived molecules that in turn may increase the effects of the generated stress.

A connection has also been observed between NO and ROS pathway under different physiological and stress conditions (Corpas et al., 2011; Groβ et al., 2013; Procházková et al., 2014). Furthermore, all components of Asa-GSH cycle have been reported to be *S*-nitrosylated (Lin et al., 2012; Tanou et al., 2012) with a different effect on protein activity (Kato et al., 2013; Begara-Morales et al., 2014, 2015).

Dehydroascorbate reductase has been identified as *S-*nitrosylation target at Cys20 under no-stress conditions in *Arabidopsis*, and this Cys20 is not over-nitrosylated under salinity or cold stress (Fares et al., 2011; Puyaubert et al., 2014). Recently, it has been reported that *S-*nitrosylation at Cys20 and Cys147 negatively regulates the enzymatic activity of DHAR in potato plants (Kato et al., 2013). Furthermore, peroxisomal recombinant pea MDAR, which has only two cysteines (Cys197 and Cys68) is also inhibited by *S*-nitrosylation (Begara-Morales et al., 2015). The authors suggest using *in silico* and evolutionary analysis that Cys68 could be the most reliable residue responsible for the loss of activity following GSNO treatment. However, future experiments such as site-directed mutagenesis and/or mass spectrometry are needed to verify this postulation. In any case, it is clear that peroxisomal pea MDAR is *S*-nitrosylated by GSNO, as corroborated by the biotin-switch method, and as result the protein activity is inhibited (Begara-Morales et al., 2015). The inhibition of DHAR and MDAR by *S-*nitrosylation (**Figure 1**) could compromise ascorbate regeneration and therefore the functioning of the cycle. Notably, in the same work it is shown that chloroplastic and cytosolic pea GR are also *S-*nitrosylated by GSNO. However, this modification does not significantly affect protein activity, as happens after tyrosine nitration (see above). In mammal cells GSNO treatment for 1h does not affect GR, although an inhibitory effect is produced after longer exposures to GSNO (Beltrán et al., 2000). In addition, human GR is inhibited by GSNO as consequence of *S-*nitrosylation of two catalytic Cys, Cys63 and/or Cys58 (Becker et al., 1995; Francescutti et al., 1996). These results suggest a different regulation of pea and mammalian GR since that pea GR activity could be unaffected by any NO-PTMs under a nitro-oxidative stress situation (Begara-Morales et al., 2015) in an attempt to maintain GSH levels and consequently the cellular redox state.

Regarding the regulation of Asa-GSH cycle by *S*-nitrosylation, the best characterized enzyme is APX. APX *S*-nitrosylation could have an essential role in physiological and stress conditions via regulation of APX activity (Correa-Aragunde et al., 2013; de Pinto et al., 2013; Begara-Morales et al., 2014), highlighting that APX can constitute a critical interface in the relationship between NO and H_2_O_2_ metabolism (Lindermayr and Durner, 2015). It has been suggested that *Arabidopsis* APX *S*-nitrosylation/denitrosylation mediated by auxins could be involved in the determination of root architecture (Correa-Aragunde et al., 2013, 2015). In this situation, APX1 is *S-*nitrosylated *in vivo* and auxins-mediated denitrosylation decreased the protein activity, an effect corroborated by the treatment of APX1 recombinant protein with CysNO (Correa-Aragunde et al., 2013). In contrast, de Pinto et al. (2013) reported that APX *S*-nitrosylation mediated by GSNO inhibits protein activity in tobacco plants and that this change could be related to programmed cell death (PCD). By *in silico* analysis, in the former study is postulated that the increase in APX activity is consequence of *S-*nitrosylation at Cys168, whereas in the latter it is suggested that the inactivation is due to *S*-nitrosylation at Cys32. However, Clark et al. (2000) reported that the inactivation of tobacco APX activity by GSNO could be due to the formation of an iron-nitrosyl complex between NO and the heme group’s iron atom. This implies that, Cys168, which is located near heme group, could be the responsible for APX activity inactivation, and not Cys32. In this sense, further experimental data (e.g., site-directed mutagenesis) could be needed to confirm what Cys is(are) involved in the (de)activation of the protein activity.

Another study described an increase in *S*-nitrosylation of pea APX as a protective mechanism in response to salinity stress (Begara-Morales et al., 2014). In this case, the cytosolic pea APX activity is stimulated by *S-*nitrosylation *in vitro* and *in vivo*. The advantage of this work is that the sequence of pea APX contains only one Cys32, making this residue the only candidate to be *S*-nitrosylated and responsible for increasing APX activity after *S-*nitrosylation. This finding has been recently corroborated by Yang et al. (2015), who showed using proteomic and mutagenesis approaches that *S*-nitrosylation at Cys32 positively regulates APX1 activity in *Arabidopsis*. In addition, they demonstrated that *S*-nitrosylation of Cys32 plays an essential role in plant response to oxidative stress and in plant immunity. As result, *S-*nitrosylation of Cys32 appears to be responsible for increasing activity of APX (**Figure 1**).

## Conclusion and Future Perspectives

Nitric oxide and H_2_O_2_ are essential signaling molecules involved in physiological processes and plant response to unfavorable conditions. These molecules share signaling pathways, so that it is not surprising to find cross-talk by which one pathway can control the function of the other. In this regard, key control points of ROS metabolism by NO are the PTMs of catalase, SODs, peroxiredoxins, and enzymes of the Asa-GSH cycle. Recent findings indicate that the antioxidant capacity of Asa-GSH cycle could be compromised under stress situations that generate nitro-oxidative stress, due to the inactivation of APX and MDAR activities by tyrosine nitration (**Figure 1**). However, APX activity is increased by *S*-nitrosylation while GR is not affected by these NO-PTMs, suggesting that GR tries to maintain GSH regeneration and therefore the cellular redox state in order to sustain the Asa-GSH cycle’s resistance to nitro-oxidative cell conditions. It bears noting that APX is under dual regulation by tyrosine nitration and *S*-nitrosylation, which are two different oxidative states related to nitro-oxidative stress. In this sense, future research should delve into the regulation of Asa-GSH cycle according to the oxidative stress generated and affected cell compartments.

## Author Contributions

The experiments were conceived and designed by: JB, FC, and JB-M. The experiments were performed by: JB-M, BS-C, MC, RV, CM-P, and MP. The data were analyzed by: JB, FC, and JB-M. The paper was written by: JB-M and JB.

## Conflict of Interest Statement

The authors declare that the research was conducted in the absence of any commercial or financial relationships that could be construed as a potential conflict of interest.

## ABBREVIATIONS

Asa-GSH cycle: ascorbate-glutathione cycle
APX: ascorbate peroxidase
DHAR: dehydroascorbate reductase
GR: glutathione reductase
MDAR: monodehydroascorbate reductase
NO-PTMs: nitric oxide-related post-translational modifications
